# Low-pressure monopolar electroresection of the prostate for glands sized > 70 vs. < 70 cc performed with continuous irrigation and suprapubic suction: perioperative and long-term outcome

**DOI:** 10.1007/s00345-017-2162-x

**Published:** 2018-01-06

**Authors:** Konrad Wilhelm, Ioana Maria Cazana, Martin Schoenthaler, Arndt Katzenwadel, Johannes Spaeth, Arkadiusz Miernik

**Affiliations:** 1 Department of Urology, University Medical Center, Hugstetterstr. 55, 79106 Freiburg, Germany; 2 Department of Anesthesiology and Critical Care, Faculty of Medicine, Medical Centre-University of Freiburg, Freiburg, Germany

**Keywords:** Prostate resection, Lower urinary tract symptoms, TUR-P, TUR syndrome, Low-pressure resection

## Abstract

**Purpose:**

To evaluate long-term efficacy and safety of low-pressure transurethral resection of the prostate for prostates < 70 cc (group 1) vs. > 70 cc (group 2).

**Patients and methods:**

In this study patients operated with monopolar TURP between 2009 and 2012 were evaluated retrospectively. During surgery a specially designed trocar (18 Fr) was placed suprapubically and connected to a suction pump to maintain stable low-pressure conditions. After sample size calculations, long-term follow-up was completed for 70 invited patients in each group up to 9/2015.

**Results:**

Follow-up period was 57 vs. 56 months for group 1 and 2, respectively (*p* = 0.56). At baseline there was no significant difference in age, IPSS, peak flow, and post void residual (PVR). Mean prostate volume was 47 cc (15–65) vs. 100 cc (70–163). Mean operating time was 55.4 vs. 82.6 min (*p* = 0.00). Blood transfusion was necessary in 0.0 vs. 2.9% (p = 0.16), and 0.0 vs. 1.4% developed TUR syndrome (*p* = 0.32). At follow-up mean relative improvement in IPSS was 63 vs. 57% (*p* = 0.29), QoL 64 vs. 64% (*p* = 0.93), peak flow 139 vs. 130% (*p* = 0.85), and PVR 58 vs. 63% (*p* = 0.80). Long-term complications included recurring adenoma in 1.4 vs. 4.3% (*p* = 0.31), and stricture in 7.2 vs. 5.8% (*p* = 0.73). 1 patient in each group reported worsening incontinence symptoms.

**Conclusions:**

In terms of safety and efficacy, the aforementioned modality of standardized monopolar TURP using suprapubic suction was non-inferior for prostates > 70 cc compared to the same procedure for prostates < 70 cc. This technique is a potential low-cost alternative for clinics that cannot afford modern laser approaches.

**Study register number:**

DRKS00006527.

## Introduction

Transurethral resection of the prostate (TURP) remains first line treatment for surgical management of lower urinary tract symptoms (LUTS) secondary to bladder outlet obstruction (BOO) caused by BPH in small and middle size glands [1, 2]. For prostates > 70 to 80 cc, the EAU guidelines recommend open prostatectomy or laser enucleation. Published studies report that TURP is associated with higher complication rates when performed in larger glands, in particular regarding blood transfusion and TUR syndrome [3]. We have carried out standardized monopolar TURP using a pressure-controlled suprapubic suction device safely and effectively in glands up to 200 cc. This study’s objective was to assess perioperative complications and long-term patient outcomes after monopolar low-pressure TUR-P in patients with glands < 70 cc vs. glands > 70 cc.

## Materials and methods

### Study design and patients

Our study was approved by the University of Freiburg Ethics Committee and performed in accordance with the ethical standards laid down in the 1964 Declaration of Helsinki and its later amendments.

The design of the study is retrospective with prospective long-term evaluation of available patients presenting for long-term follow-up after invitation. We identified patients who underwent transurethral resection of the prostate after 1/2009 (the beginning of electronic patient charts in our department) by reviewing our charts retrospectively. Until 2014 no laser treatment was performed in our clinic. Open prostatectomy was discussed with patients with large glands > 100 cc but most patients underwent transurethral resection after informed consent.

### Sample size calculation

Ten patients with prostate volume < 70 cc (group 1) and 10 with prostate volume > 70 cc (group 2) were reviewed initially. Based on those patients, we did a power calculation for a non-inferiority study, with the percentage of IPSS-improvement as primary outcome and a 15% non-inferiority-margin, resulting in a sample size of 69 patients per group to yield 80% power.

Consequently, patients with initial prostate size < 70 cc as measured by transrectal sonography and those with bigger glands were identified and a invitation letter was sent to invite them for a long-term follow-up visit and possible inclusion in the study. Interested patients presented for a follow-up visit. At follow-up they gave informed consent to be included in the study. Exclusion criteria for the study were known prostate cancer before the TUR-P and no available long-term follow-up or informed consent. Patients with incidental carcinoma in the resected material remained in the study. Patients with a preoperatively indwelling catheter were included for our assessment of subjective parameters and complications, but were excluded from the *Q*_max_ and PVR evaluations.

The study was closed when the calculated sample size was reached.

### Surgical technique

All surgeons are experienced board-certified surgeons or advanced residents under the supervision of board-certified surgeons.

**Fig. 1:**
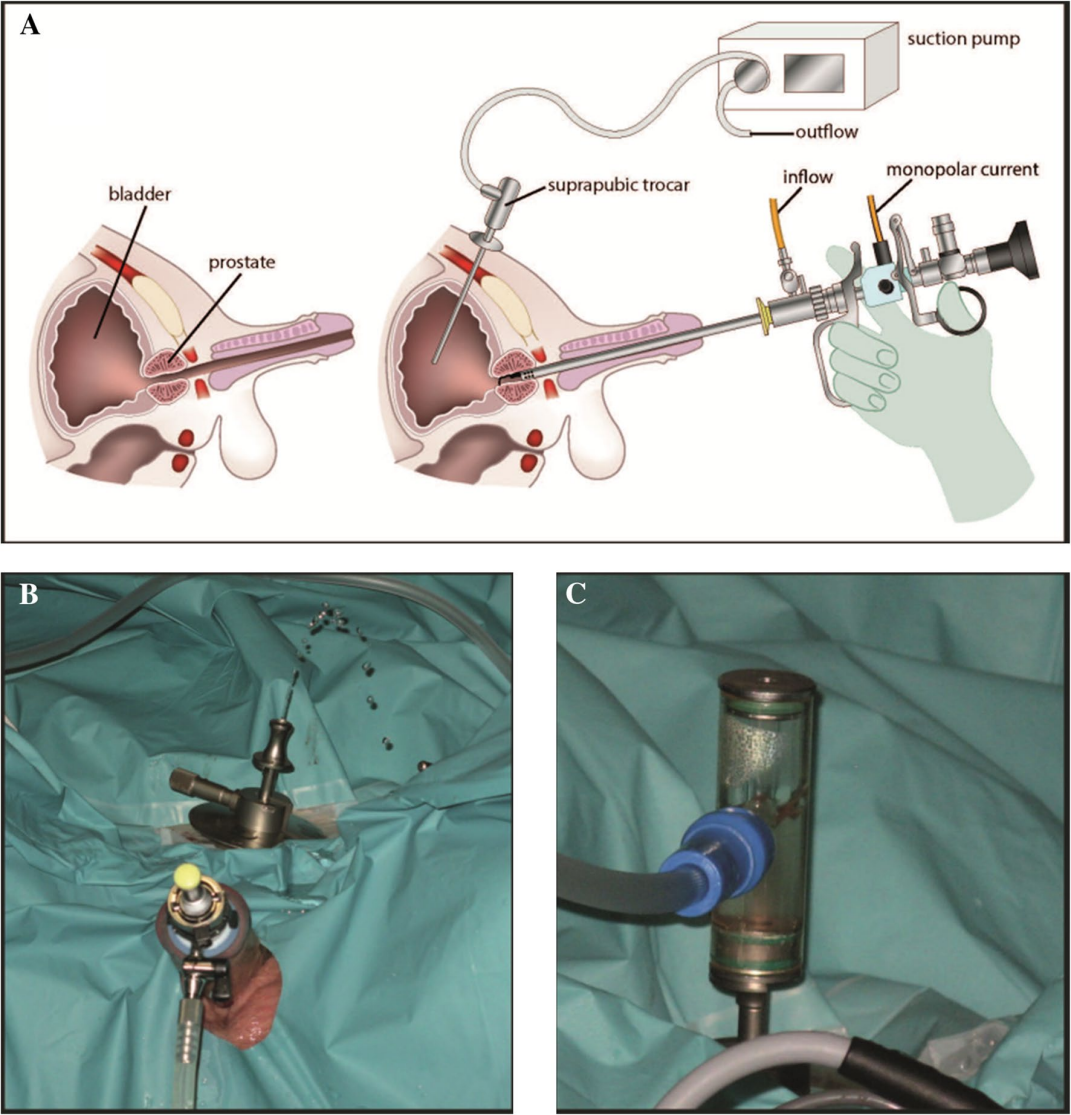
**a** Scheme of equipment. The suprapubic trocar is connected to a suction hose and pump. **b** Suprapubic suction trocar. This shows the suprapubic suction trocar directly after insertion into the previously filled bladder. The jet of irrigation fluid confirms the correct placement in the bladder. **c** Suprapubic suction trocar 2. The inner sheath of the suction trocar is placed and connected to a suction hose in order to maintain constant low-pressure conditions of irrigation fluid

Monopolar TURP is performed in highly standardized fashion in dorsal lithotomy position under general or spinal anesthesia. After the cystoscopic exclusion of bladder tumors, the bladder is filled with purisole (mannitol 5% solution in 3000 ml bags). A specially designed “flow controller” trocar 18 Fr (Olympus, Hamburg, Germany, first described by Korth in 1989 [4, 5]) is placed suprapubically and connected to a suction pump to maintain stable low-pressure conditions of irrigation fluid (about 14–18 cmH_2_O) (Fig. 1). TURP is then performed with a resectoscope (Olympus, 24 Fr) with one inflow channel. The instrument itself is not a continuous flow instrument but due to the permanent outflow via the suprapubic trocar continuous irrigation is possible. At the end of the procedure, the suprapubic trocar is removed and the puncture site is coagulated to avoid bleeding. A transurethral foley catheter is put in place and continuous irrigation maintained until the urine is clear.

### Assessment

Baseline data at the time of surgery were collected including age, BMI, ASA [6], PSA, prostate size (measured by transrectal ultrasound), IPSS, QoL, ICIQ, IIEF, PVR, and the *Q*_max_ and Charlson comorbidity indices [7]. Perioperative data included resection weight, histologic results, length of catheterization and complications including transfusion rates, TUR syndrome as mentioned in the patient chart, and complications attributable to the suprapubic puncture. Functional outcomes were monitored at the latest available follow-up.

Early complications during the first 30 days after the surgery and late complications such as surgical procedures for recurrent adenoma, stricture or bladder neck obstruction were assessed. Complications are reported according to the Clavien classification modified for TURP [7].

### Statistics

Changes from baseline for IPSS, QoL, *Q*_max_, PVR, etc. compared to the latest available follow-up time and differences between the two groups were calculated using a Student’s two-tailed *t* test. Statistical significance was defined as *p* < 0.05. Statistics were performed using IBM SPSS Statistics 22.

## Results

### Patient characteristics

Between 1/2014 and 9/2015, a total of 139 patients followed the invitation for a long-term follow-up and agreed to be enrolled in the study, of whom 69 and 70 had initial prostate size < 70 and > 70 cc, respectively. Patient characteristics are shown in Table 1. Six and 14 patients in group 1 and 2 had an indwelling catheter prior to TURP, respectively. 12 patients in each group were on ASS therapy, which was not interrupted for the intervention. 6 vs. 5 were on warfarin therapy, which we bridged with low molecular heparin for the intervention.

**Table 1:** Patient characteristics

	Small prostates (< 70 cc) *N* = 69				Large prostates (> 70 cc) *N* = 70				*p* value
	Mean	Median	SD	Range	Mean	Median	SD	Range	
Age (years)	67.3	68	6.30	50–79	69.4	70	6.3	55–87	0.052
BMI	27.06	26.72	3.19	20.76–37.37	26.77	26.62	3.69	19.57–37.03	0.631
ASA	2	2	1	1–3	2	2	1	3	0.228
CCI	0.7	0	1	0–4	0.8	0	1.3	0–6	0.808
Prostate Volume (cc)	47	50	12.8	15–65	100.2	97.5	23.1	70–163	0
PSA (ng/ml)	3.26	2.49	2.72	0.26–14.81	7.89	6.23	6.12	1.05–31.70	0

*ASA* American Society of Anesthesiologists physical status classification, *CCI* Charlson Comorbidity Index

### Perioperative results

Procedure characteristics are shown in Table 2. Operative time was significantly longer in group 2, but catheterization time and length of hospital stay did not differ between groups 1 and 2.

**Table 2:** Procedure characteristics

	Small prostates (< 70 cc)				Large prostates (> 70 cc)				*p* value
	Mean	Median	SD	Range	Mean	Median	SD	Range	
Operative time (min)	55.4	48	26.5	17–135	82.6	75.5	37.7	32–230	0.000
Resection weight (g)	24.87	24	10.4	5–60	54.35	50	27.3	35–170	0.000
Catheterization time (h)	48	48	4.1	24–72	50.1	48	7.9	48–96	0.057
Length of stay (h)	78.6	72	31	48–288	78.5	72	17.3	48–168	0.982

### Functional results at long-term follow-up

Mean follow-up was 57 vs. 56 months for group 1 and 2, respectively.

Paired outcome measurements for our functional results are shown in Tables 3 and 4. We observed a highly significant improvement from baseline to follow-up in both groups regarding all the tested items. There was no significant difference between the two groups.

**Table 3:** Paired outcome measurements subjective

	Small prostates (< 70 cc)	Large prostates (> 70 cc)
IPSS		
*N*	66	57
Follow-up months	55	56
BL, mean (SD)	19.8(7.5)	17.2 (8)
FU, mean (SD)	7.26 (5.86)	6.22 (6.29)
Δ, mean (SD)	− 13.00 (7.68)	− 11.07 (932)
Relative Δ %, mean	− 62.87	55.98
*p* value absolute Δ	0.210	
*p* value relative Δ	0.287	
IPSS QoL		
*N*	65	65
Follow-up months	57	56
BL, mean (SD)	4.01 (1.38)	3.54 (1.39)
FU, mean (SD)	1.42 (1.29)	1.09 (0.99)
Δ, mean (SD)	− 2.78 (1.85)	− 2.48 (1.66)
Relative Δ %, mean	− 64.41	− 63.80
*p* value absolute Δ	0.359	
*p* value relative Δ	0.927	
ICIQ		
*N*	45	45
Follow-up months	58	54
BL, mean (SD)	4.33 (4.59)	4.25 (5.02)
FU, mean (SD)	2.17 (3.65)	1.58 (3.39)
Δ, mean (SD)	− 1.99 (4.74)	− 1.91 (4.43)
Relative Δ %, mean	–	–
*p* value absolute Δ	0.897	
*p* value relative Δ	–	

The difference of the *N* is explained by the fact that not all patients provided all the answers in the questionnaires. Patients with incomplete questionnaires were not included in the analysis of subjective outcomes

*BL* base line value, *FU* follow-up value, *Δ* absolute difference FU − BL, *Relative Δ* percentage improvement

**Table 4:** Paired outcome measurements objective

	Small prostates (< 70 cc)	Large prostates (> 70 cc)
*Q*_max_		
*N*	50	53
Follow-up months	53	55
BL, mean (SD)	11.43 ml/s (5.42)	11.52 ml/s (5.23)
FU, mean (SD)	19.46 ml/s (11.02)	22.11 ml/s (10.79)
Δ, mean (SD)	9.8 ml/s (10.45)	10.84 ml/s (11.16)
Relative Δ, mean	139.20%	129.77
*p* value absolute Δ	0.694	
*p* value relative Δ	0.850	
PVR		
*N*	59	57
Follow-up months	57	56
BL, mean (SD)	109.12 ml (77.08)	151.86 ml (167.30)
FU, mean (SD)	34.84 ml (40.29)	25.98 ml (56.66)
Δ, mean (SD)	− 70.56 ml (79.86)	− 122.42 ml (164.97)
Relative Δ, mean	− 58.35%	− 62.53%
*p* value absolute Δ	0.099	
*p* value relative Δ	0.801	

The difference of the *N* is explained by the fact that not all patients provided all the answers in the questionnaires. Patients with incomplete questionnaires were not included in the analysis of subjective outcomes

*BL* base line value, *FU* follow-up value, *Δ* absolute difference FU − BL, *Relative Δ* percentage improvement

### Complications

Short-term, long-term, and overall complications are illustrated in Table 5; specific complications are shown in Table 6.

**Table 5:** Complications

	Small prostates (< 70 cc) *N* = 69	Large prostates (> 70 cc) *N* = 70
Short term complications		
Clavien 1	21 (30.43%)	22 (31.88%)
Clavien 2	2 (2.90%)	9 (13.04%)
Clavien 3a	13 (18.84%)	7 (10.14%)
Clavien 3b	8 (11.59%)	11 (15.94%)
Clavien 4	0	0
Clavien 5	0	
Long-term complications		
Clavien 1	0	0
Clavien 2	0	0
Clavien 3a	0	0
Clavien 3b	8 ((11.59%)	5 (7.25%)
Clavien 4	0	0
Clavien 5	0	0
Overall		
Clavien 1	22 (31.88%)	23 (33.33%)
Clavien 2	3 (4.35%)	10 (14.49%)
Clavien 3a	10 (14.49%)	5 (7.25%)
Clavien 3b	13 (18.84%)	17 (24.64%)
Clavien 4	0	0
Clavien 5	0	0

The complications were attributed to the Clavien score modified for TUR-P (citation see manuscript)

**Table 6:** Complications detailed

	Small prostates (< 70 cc)	Large prostates (> 70 cc)	*p*
Short-term complications			
*N*	69	70	
Transient hematuria	46 (67%)	46 (67%)	0.906
Bleeding requiring surgical revision	8 (11.6%)	11 (15.7%)	0.483
Bleeding requiring blood transfusions	0 (0%)	2 (2.9%)	0.160
Failed voiding trial	4 (5.8%)	3 (4.3%)	0.442
Positive urine culture postoperative	2 (2.9%)	9 (12.9%)	0.030*
Fever	1 (1.4%)	2 (2.9%)	0.571
Urosepsis	0 (0%)	0 (0%)	–
TUR-syndrome	0 (0%)	1 (1.4%)	0.323
Pulmonary thromboembolism	0 (0%)	0 (0%)	–
Myocardial infarction	0 (0%)	0 (0)	–
Death	0 (0%)	0 (0%)	–
Long-term complications			
Follow-up months	57	56	
Bladder neck contracture	4 (5.9%)	1 (1.4%)	0.169
Urethral stricture	5 (7.2%)	4 (5.8%)	0.733
Residual/recurrent adenoma needing surgical treatment	1 (1.4%)	3 (4.3%)	0.314

Differences between the two groups were calculated using a Student’s two-tailed* t* test.

*Statistical significance was defined as* p* < 0.05

The most frequent short-term complication (first 30 days postoperative) was transient hematuria, self-limiting in most patients. Surgical revision due to prolonged bleeding was needed in 8 (11.6%) vs. 11 (15.7%) patients in group 1 vs. 2, respectively (*p* = 0.483). Blood transfusions were necessary in 0 vs. 2 (2.9%) patients (*p* = 0.160).

One patient in group 2 was diagnosed as having TUR syndrome. One patient in group 2 suffered a myocardial infarction during the first 30 days after surgery.

A positive urine culture during the first 3 days after surgery was significantly more frequent in group 2 (2 vs. 9 patients, *p* = 0.030*), but did not result in higher rates of fever or sepsis.

Regarding long-term complications, we noted a tendency for more bladder neck contractures in group 1, but more recurrent adenomas in group 2. Strictures revealed no difference.

No patient reported new onset incontinence related to the surgery.

### Patient satisfaction

Patient satisfaction was measured via the Freiburg Index for Patient Satisfaction [8, 9]; patients in both groups rated the surgical intervention as “very good” to “good” (1.78 vs. 1.67, *p* = 0.6).

### Pain and analgesic consumption

Pain and analgesic consumption were assessed using the visual analogue scale (VAS) and the cumulative analgesic consumption score [10] for day 0, 1, and 2 after surgery (Table 7). This score allows a more objective assessment of perioperative pain than the subjective visual analogue scale. No difference was observed in the mean VAS score; group 2’s analgesic consumption was slightly lower.

**Table 7:** Analgesic consumption

	Small prostates (< 70 cc)	Large prostates (> 70 cc)	*p* value
Day 0			
VAS	1.36	1.07	0.332
CACS	6.32	5.37	0.033
Day 1			
VAS	0.42	0.39	0.847
CACS	0.67	0.50	0.434
Day 2			
VAS	0.36	0.31	0.789
CACS	0.68	0.53	0.543
Total			
VAS	0.86	0.70	0.464
CACS	7.71	6.13	0.018

Pain and analgesic consumption were assessed using the visual analogue scale (VAS) and the cumulative analgesic consumption score for day 0, 1, and 2 after surgery. This score allows a more objective assessment of perioperative pain than the subjective visual analogue scale and is explained in detail in the article cited in the manuscript

*VAS* visual analogue scale, *CACS* cumulative analgesic consumption score

## Discussion

TURP is still the recommended first line treatment for BPH with gland sizes between 30 and 70–80 ml [1]. It is a safe and effective treatment option and improves relevant patient outcomes significantly: a meta-analysis revealed an IPSS reduction of 70%, *Q*_max_ + 162%, and PVR urine − 77% [11]. However, long operating times with hypo-osmolar irrigation fluid as needed for monopolar TURP can raise the risk for TUR-syndrome [12] or bleeding [13].

Alternatives like open prostatectomy, holmium laser enucleation or laser vaporization of the prostate are, therefore, recommended for larger glands. Open prostatectomy is an effective alternative, but due to its safety profile (blood transfusion rates 7–14%), it is considered an invasive option [13, 14]. Holmium laser enucleation is an accepted alternative for treating obstructing glands > 70 to 80 cc, but requires special equipment and a high-power laser [15].

The procedure we describe here is not new. Korth described this technique in 1989, and Heidler published an RCT comparing the Korth trocar with another suprapubic drainage system and the well-known Iglesias continuous-flow resectoscope [4, 16]. They showed that intravesical pressure is diminished with the trocar, and that this helps to keep plasma sodium concentrations stable. However, this technique has not gained acceptance and is rarely used. We identified no study applying this technique for prostates > 70 g and believe that we demonstrate hereby a means of overcoming the irrigation-related problems in monopolar TUR-P without requiring invasive open prostatectomy or expensive new equipment.

### Study design

To adequately investigate prostate size as an influencing factor, we decided to compare small prostates vs. large prostates, all operated with the same technique. Randomization is not possible with this design. We chose 70 cc as the cut-off in accordance with other publications [14, 17]. Despite our having a mixed retrospective–prospective study design, our study’s patients exhibited no significant differences in baseline characteristics like age and comorbidities. Their follow-up periods were also the same.

### Procedure characteristics

As expected, group 2’s operating time was longer. It has been postulated that resection times over 90 min enhance the risk for irrigation-related complications [18]. The mean resection time of 82 min for the bigger glands in this series is still below this (virtual) limit. Nevertheless, even patients requiring longer operation times experienced no TUR syndrome or excessive bleeding. The resection weight in group 2 is lower than expected. The most probable explanation is a combination of imprecise sonographic measurement of prostate size, dehydration of resected material before weighing, and incomplete removal of adenoma.

### Functional results

Our patients’ functional results regarding smaller and bigger prostates (IPSS, QoL, ICIQ, PVR, QoL) at long-term follow-up are the same and in line with other data on monopolar TUR-P [11, 19, 20]. A limitation of our study is that we could not assess data at more time points sooner after the intervention.

### Short-term complications

Our rates for failed voiding trials and blood transfusions correspond to those reported in the literature [20, 21].

Group 2 exhibited a significantly higher rate of positive urine cultures requiring antibiotic coverage, but no severe infectious problems were evident.

### Bleeding

Bleeding was reported to be more likely with a resection time > 90 min (7.3 vs. 0.9%) [18].

We noted two blood transfusions in group 2 vs. 0 in group 1 (not significant in our series), but not significantly more bleeding scenarios requiring surgical intervention. Patients needing such revision were mainly patients with heparin bridging of warfarin therapy. Our bleeding percentages are still much lower than those reported in the literature.

### TUR syndrome

Dilutional hyponatremia is a feared complication of monopolar transurethral resection with hypoosmolar irrigation fluid, resulting in neurologic and cardiologic symptoms. There is evidence that the incidence of developing TUR syndrome is significantly higher (2%) in patients whose resection time is > 90 min compared to those whose resection time lasts under 90 min (0.7%) [18]. In our series, only one TUR syndrome was diagnosed in group 2—thus demonstrating the suprapubic suction pump’s effectiveness. As a limitation it must be stated that the postoperative course of TUR-P patients did only include measurement of serum sodium in cases of clinical suspicion of TUR syndrome.

It is noteworthy that we observed no complications related to the suprapubic trocar in this series.

### Long-term complications

We detected no statistical differences in the rate of reintervention because of bladder neck contracture, urethral stricture, or residual adenoma. The percentages presented here seem to be acceptable, especially when our long follow-up (nearly 5 years) is considered. However, the percentage of reinterventions was higher in the group with large glands and it could be possible that with longer follow-up and larger patient numbers this could become statistically significant.

This technique might appear outdated. It was first described by Korth and is based on even older publications recommending suprapubic suction devices to maintain low-pressure conditions in the bladder [4, 5, 22]. As a consequence of the spread of industry-driven technological progress mandating alternatives such as laser techniques, many investigations have been conducted, although many of the more recent ones revealed no overwhelming advantages over TUR-P, merely non-inferiority [20, 21]. In our department we introduced HoLEP for bigger glands, and we now employ it in conjunction with almost every surgical BPH therapy regardless of the prostate volume.

However, we aimed to remind the urologic community of this effective, safe, and cheap alternative to standard treatment options for bigger prostates. We believe it is a potentially promising option in clinical settings where open prostatectomy seems overly invasive but where introducing HoLEP or other laser methods is too expensive. To the best of our knowledge, this is the first study to compare this low-pressure monopolar resection for patients with LUTS secondary to BPH with small vs. large prostatic volumes with such long follow-up.

The study is limited by its retrospective character. We cannot provide short-term functional results because not all our patients presented for follow-up at our department.

There is a risk of bias because some patients with worse outcomes might have refused to participate in the long-term follow-up. Postoperative blood counts are not available for all patients, because we require postoperative blood analyses only from patients presenting prolonged bleeding or suspected anemia.

Furthermore, some patients with bigger glands might have been operated on by more experienced surgeons, thus creating a performance bias. This study was powered for IPSS-improvement. The minimal differences in complications we observed here might have become statistically significant had we enrolled more patients.

No direct comparison has been made with patients treated by endoscopic enucleation or open simple prostatectomy.

## Conclusion

In terms of safety and efficacy, the present modality of standardized monopolar TURP using suprapubic suction was non-inferior for prostates > 70 cc compared to the same procedure for prostates < 70 cc. These findings add new evidence on affordable surgical treatment options for large prostatic glands. However, this technique requires additional validation in larger cohorts and at other centers, and direct comparison to more recent laser ablative interventions.

## Abbreviations

ASA: American Society of Anesthesiologists physical status classification
BMI: Body mass index
BOO: Bladder outlet obstruction
BPH: Benign prostate hyperplasia
CCI: Charlson Comorbidity Index
EAU: European Association of Urology
HoLEP: Holmium laser enucleation of the prostate
IPSS: International prostate symptom score
LUTS: Lower urinary tract symptoms
PVR: Post-void residual urine
*Q*_max_: Maximum uroflow
QoL: Quality of life
RCT: Randomized controlled trial
TURP: Transurethral resection of the prostate
